# T cell-tropic HIV efficiently infects alveolar macrophages through contact with infected CD4+ T cells

**DOI:** 10.1038/s41598-021-82066-x

**Published:** 2021-02-16

**Authors:** Abigail E. Schiff, Alice H. Linder, Shillah N. Luhembo, Stephanie Banning, Martin J. Deymier, Thomas J. Diefenbach, Amy K. Dickey, Athe M. Tsibris, Alejandro B. Balazs, Josalyn L. Cho, Benjamin D. Medoff, Gerhard Walzl, Robert J. Wilkinson, Wendy A. Burgers, Björn Corleis, Douglas S. Kwon

**Affiliations:** 1 Ragon Institute of MGH, MIT, and Harvard, Massachusetts General Hospital, Harvard Medical School, Cambridge, MA USA; 2 Harvard Medical School, Boston, MA USA; 3 Division of Infectious Diseases, Brigham and Women’s Hospital, Harvard Medical School, Boston, MA USA; 4 Division of Pulmonary and Critical Care Medicine, Massachusetts General Hospital, Boston, MA USA; 5 DST-NRF Center of Excellence for Biomedical Tuberculosis Research, South African Medical Research Council Centre for Tuberculosis Research, Division of Molecular Biology and Human Genetics, Faculty of Medicine and Health Sciences, Stellenbosch University, Cape Town, South Africa; 6 Wellcome Center for Infectious Diseases Research in Africa and Institute of Infectious Disease and Molecular Medicine, University of Cape Town, Observatory, 7925 Republic of South Africa; 7 Department of Infectious Disease, Imperial College London, London, W12 ONN UK; 8 The Francis Crick Institute, 1 Midland Road, London, NW1 AT UK; 9 Division of Medical Virology, Department of Pathology, University of Cape Town, Cape Town, Republic of South Africa; 10 Division of Infectious Diseases, Massachusetts General Hospital, Boston, MA USA; 11 Division of Pulmonary, Critical Care and Occupational Medicine, University of Iowa, Iowa City, IA USA; 12 Institute of Immunology, Friedrich-Loeffler-Institute, Federal Research Institute for Animal Health, Greifswald, Isle of Riems Germany

**Keywords:** Virology, Retrovirus, Virus-host interactions, HIV infections, Mucosal immunology, Monocytes and macrophages

## Abstract

Alveolar macrophages (AMs) are critical for defense against airborne pathogens and AM dysfunction is thought to contribute to the increased burden of pulmonary infections observed in individuals living with HIV-1 (HIV). While HIV nucleic acids have been detected in AMs early in infection, circulating HIV during acute and chronic infection is usually CCR5 T cell-tropic (T-tropic) and enters macrophages inefficiently in vitro. The mechanism by which T-tropic viruses infect AMs remains unknown. We collected AMs by bronchoscopy performed in HIV-infected, antiretroviral therapy (ART)-naive and uninfected subjects. We found that viral constructs made with primary HIV envelope sequences isolated from both AMs and plasma were T-tropic and inefficiently infected macrophages. However, these isolates productively infected macrophages when co-cultured with HIV-infected CD4+ T cells. In addition, we provide evidence that T-tropic HIV is transmitted from infected CD4+ T cells to the AM cytosol. We conclude that AM-derived HIV isolates are T-tropic and can enter macrophages through contact with an infected CD4+ T cell, which results in productive infection of AMs. CD4+ T cell-dependent entry of HIV into AMs helps explain the presence of HIV in AMs despite inefficient cell-free infection, and may contribute to AM dysfunction in people living with HIV.

## Introduction

Individuals with untreated HIV-1 (HIV) are at increased risk of pulmonary infections with mycobacteria and other bacteria, viruses, and fungi^1–3^. The first line of defense against these infections in the lung are alveolar macrophages (AMs), which comprise the vast majority of immune cells in the alveolar space^4,5^. AMs from individuals with untreated HIV are dysfunctional, with impaired phagocytic activity^6–8^, proteolytic activity^9^, and bacterial killing^10^. The lung is an early site of HIV and simian immunodeficiency virus (SIV) replication in humans, humanized mice and non-human primates (NHPs)^11–15^, and HIV nucleic acids are detectable in AMs in both antiretroviral (ART)-naive and treated individuals^9,16–22^. Thus, HIV is found in the lung and in AMs early in the course of HIV-1 infection, but the mechanism of entry of HIV into AMs remains incompletely understood.

Cell tropism in HIV infection is determined by the coreceptor usage and affinity of the envelope (Env) surface protein for host CD4^23^. These phenotypes are separate, with coreceptor usage determined by the Env V3 region and macrophage or T cell tropism (M- or T-tropism, respectively) determined by the Env CD4 binding site^23,24^. There are three common combinations of coreceptor usage and cellular tropism. The first is CCR5-utilizing T-tropic HIV, which accounts for transmitted/founder viruses and most circulating viruses^24,25^. The second, CXCR4-utilizing T-tropic HIV, arises late in infection in about 50% of people living with HIV^26^. The third, M-tropic HIV, can use CCR5, CXCR4 or both coreceptors, and arises in some individuals late in infection and in T cell-depleted environments such as in advanced HIV-1 disease, in the central nervous system, or in settings of experimental depletion of CD4+ T cells^27–29^. The main target cells in early infection are mucosal CD4+ T cells, which are activated, have high expression levels of both CD4 and CCR5, and are depleted during HIV infection^15,30,31^. Monocyte-derived macrophages (MDMs) have approximately 20-fold lower surface expression of CD4 than CD4+ T cells^32–34^, and T-tropic HIV strains enter^33^ and replicate^35^ inefficiently in MDMs. Importantly, M- or T-tropism can be recapitulated by cloning of specific *env* genes into a replication-competent backbone, demonstrating that Env is responsible for this tropism^25^. CD4 and CCR5 surface expression and HIV infection rates can vary widely in MDMs depending on the conditions used for their maturation^34^, and HIV receptor surface expression and infection rates may be different compared to tissue resident macrophages like AMs^36,37^. Therefore, it is important to understand M- and T-tropic HIV entry and infection rates of primary AMs. Prior reports have shown that AMs are susceptible to M-tropic HIV entry and replication in vitro^38–40^. However, no study has established the tropism of HIV isolated from AMs from HIV-infected individuals, nor has any study established a mechanism of AM infection by T-tropic HIV strains, which predominate during acute and chronic stages of infection.

Evidence from humanized mice and NHPs infected with T-tropic HIV or SIV shows that infection of tissue macrophages in the spleen, central nervous system and lymph nodes may be dependent on the presence of CD4+ T cells^41–43^. Humanized mice reconstituted with human macrophages and not with human lymphocytes can be infected with M-tropic but not T-tropic HIV strains^41^. In NHPs, T-tropic SIV DNA is only measurable in macrophages residing in lymphoid tissues that have high numbers of CD4+ T cells, including the spleen and mesenteric lymph nodes^43^. A possible explanation for these observations is that interactions between macrophages and infected CD4+ T cells are needed to achieve high levels of macrophage infection. Prior studies suggest that T-tropic HIV inefficiently infects MDMs in vitro^25^, and while M-tropic HIV can infect macrophages, this is more efficient through contact with infected CD4+ T cells^44,45^. HIV transmission to AMs through T cells has not been studied in human AMs.

## Results

### HIV can be detected in alveolar macrophages (AMs) from individuals with untreated HIV infection

**Figure 1:**
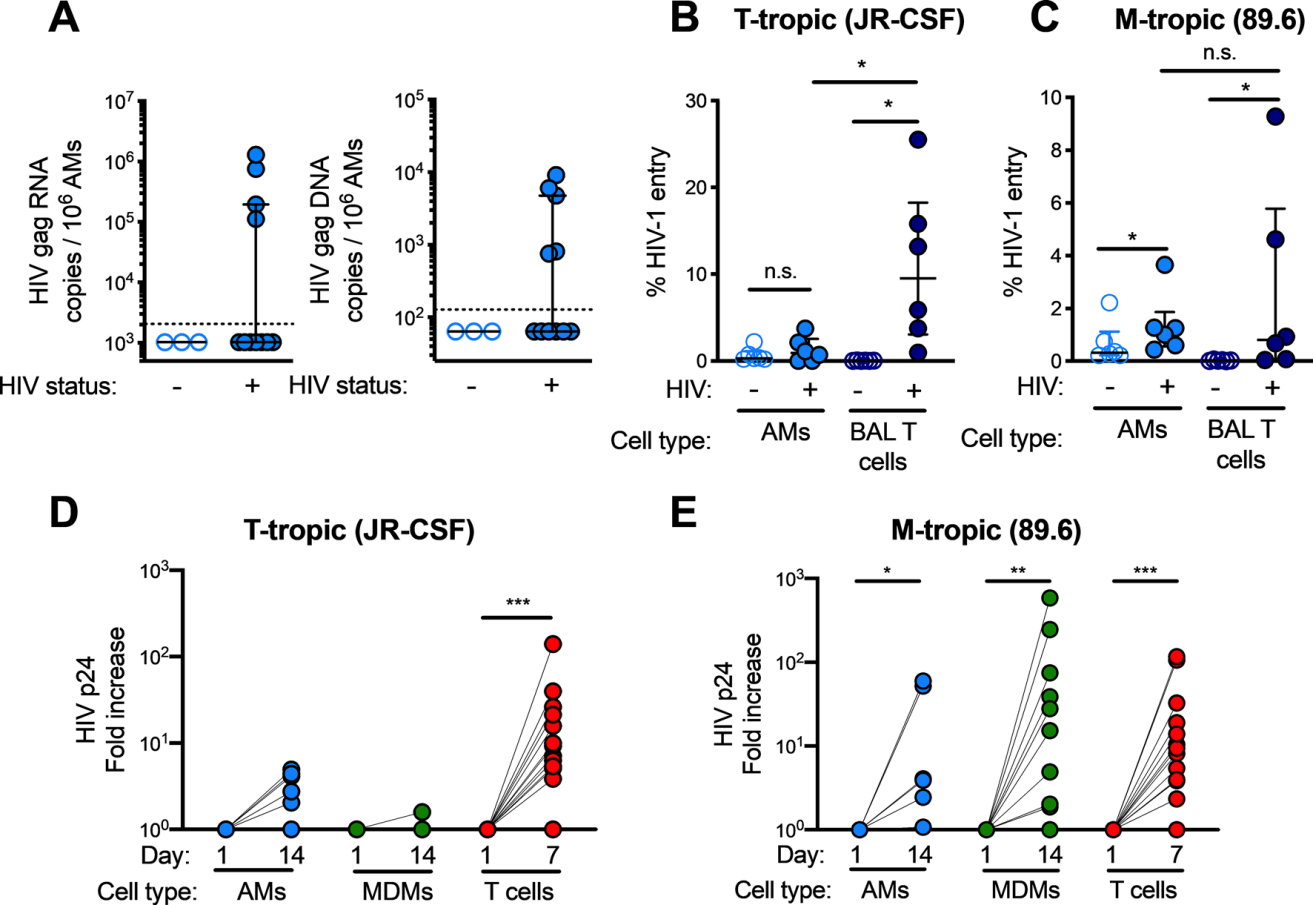
HIV RNA and DNA can be detected in AMs despite low T-tropic HIV entry and replication rates. (**A**) HIV Gag RNA and DNA are detectable in alveolar macrophages (AMs) from individuals with untreated HIV infection. HIV Gag RNA or DNA copies per cell by qRT-PCR in AMs from the Cape Town cohort. Dashed lines denote the limit of detection for the assay, and negative values are plotted at 50% of the limit of detection. (**B**, **C**) Viral entry was detected in AMs and BAL CD4+ T cells from HIV-uninfected subjects using gravity infection for 12 h with a BLaM-Vpr construct (HIV+) of JR-CSF (**B**) or 89.6 (**C**) or mock infection (HIV-) at a multiplicity of infection (MOI) of 1. HIV entry was detected by cleavage of a fluorescent BLaM substrate and measured by flow cytometry; n = 6. (**D**, **E**) CD4+ T cells, MDMs and AMs were infected with replication-competent JR-CSF (**D**) or 89.6 (**E**) at a MOI of 0.2 for 12 h. HIV p24 levels in the supernatant were measured by ELISA at the indicated time points and normalized to input at day 1; n = 11 for T cells, n = 12 for MDMs, and n = 6 for AMs. Statistics were done by Kruskal–Wallis tests with Dunn’s multiple test correction in B and C. *p < 0.05, **p < 0.01, ***p < 0.001.

To determine levels of HIV nucleic acids in AMs from HIV-infected individuals, we performed bronchoscopies with bronchoalveolar lavage (BAL) in a cohort of HIV-infected ART naive and uninfected individuals living in Cape Town, South Africa (Table 1). All the participants in the cohort had evidence of immune sensitization to *Mycobacterium tuberculosis* but no indications of active disease. Cells from each bronchoscopy were adhered and non-adherent cells were removed; 99–100% of the remaining cells were AMs by microscopy (Supplementary Table 1). Using qPCR for HIV Gag, we detected Gag RNA in purified AMs from 4/11 (36.4%) participants and Gag DNA in AMs from 5/11 (45.5%) participants (Fig. 1A). While HIV RNA was not detected in all individuals, the BAL viral load was significantly higher in participants with detectable HIV RNA in the corresponding AM sample (Supplementary Fig. 1A). HIV RNA and DNA were both detected in samples from two participants, while two other participants had detectable HIV RNA only and three had detectable HIV DNA only. Overall, these findings were similar to prior reports using AMs from ART-naive participants in which HIV RNA and DNA was detectable in AMs from a median of 38.7% and 62.6% individuals, respectively, although the values varied widely across studies^18,20,21,40,46–50^. These data demonstrate that a significant number of HIV-infected individuals harbor AMs with detectable HIV RNA and DNA.

**Table 1:** Characteristics of participants in the Cape Town cohort.

	Healthy control	Viremic HIV
Number (male/female)	14 (5/9)	15 (2/13)
Age	24.5 (21–26.75)	32 (30–34.5)*
Smokers	35.70%	0%*
Plasma viral load (copies per mL)	N/A	6383 (619–12,097)
BAL viral load (copies per mL ELF)	N/A	5546 (1036–28,034)
CD4 + T cells per μl	902 (801–1159)	571 (544–676)*
Purity of adhered AMs (%)	100 (99–100)	99 (98–100)

All participants were antiretroviral therapy-naive and were sensitized to *Mycobacterium* *tuberculosis* as determined by QuantiFERON TB Gold test without clinical or radiographic evidence of active TB. BAL, bronchoalveolar lavage; ELF, epithelial lining fluid; AM, alveolar macrophage. Values are expressed as median with interquartile range. Statistical analysis was performed with Mann–Whitney test, except for smokers, where Fisher’s exact test was performed. *p < 0.05 compared to healthy controls.

### Cell free T-tropic HIV inefficiently enters and replicates in MDMs and AMs

Since most circulating HIV in acute and chronically infected individuals is T-tropic, we compared the entry and replication capacity of T-tropic and M-tropic HIV in AMs and MDMs from HIV-uninfected individuals. We first compared HIV receptor expression on AMs, MDMs, and BAL CD4+ T cells and observed that fewer AMs expressed CD4 and CCR5 compared to MDMs and BAL CD4+ T cells (Supplementary Fig. 1B and C). Using a beta-lactamase (BLaM)-Vpr reporter assay that measures viral entry, we found that the CCR5-using T-tropic HIV strain JR-CSF did not significantly enter AMs, but was able to enter BAL CD4+ T cells, suggesting that BAL CD4+ T cells are more permissive targets of T-tropic HIV entry in the lungs (Fig. 1B). This entry into BAL CD4+ T cells was blocked by the CCR5 inhibitor maraviroc (Supplementary Fig. 1D). Using the CCR5 and CXCR4-utilizing M-tropic HIV strain 89.6, in contrast, both AMs and BAL CD4+ T cells allowed viral entry (Fig. 1C). Next, we utilized replication competent T-tropic and M-tropic HIV and showed that T-tropic HIV JR-CSF replicated in blood CD4+ T cells but not in MDMs or AMs (Fig. 1D and Supplementary Fig. 1E). M-tropic HIV 89.6, on the other hand, replicated in blood CD4+ T cells, MDMs, and AMs (Fig. 1E and Supplementary Fig. 1F). These experiments indicate that relatively few AMs express HIV receptors and that HIV infection by a lab-adapted CCR5-using T-tropic HIV strain, which is similar to those that predominate in early stages of infection, inefficiently enters and replicates in AMs and MDMs.

### AM- and plasma-derived HIV is T-tropic

**Figure 2:**
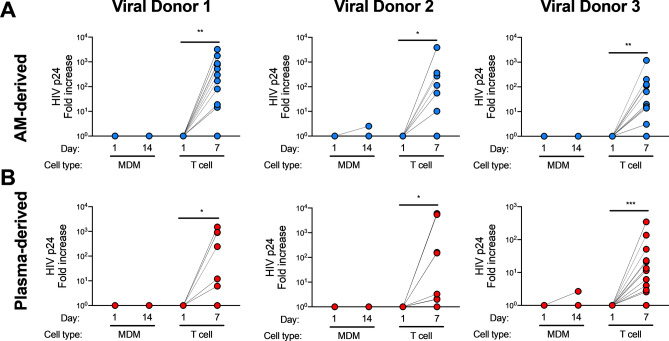
AM- and plasma-derived HIV primary isolates are T-tropic. HIV *env* isolated from AMs or plasma from 3 HIV-infected donors was cloned into an HIV NL4-3 *env* deleted backbone plasmid. Replication-competent virus was added to activated CD4+ T cells or MDMs for 12 h and washed, and supernatant was collected 1 h after washing (Day 1) and after 7 or 14 days. Data is shown as fold change of HIV p24 in supernatant at the final timepoint (Day 7 for T cells, Day 14 for MDMs) compared to Day 1. Cells were infected with AM-derived viral isolates (**A**) from viral donor 1, 2 or 3 or plasma-derived viral isolates (**B**) from viral donor 1, 2 or 3. Data from all viral isolates from the same viral donor and anatomical site were pooled. CD4 + T cells and MDMs were isolated from six HIV-uninfected blood donors. In (**A**), n = 12 (6 cell donors infected with 2 viral isolates) for viral donor 1, n = 18 (6 cell donors infected with 3 viral isolates) for viral donor 2, and n = 18 (6 cell donors infected with 3 viral isolates) for viral donor 3; in (**B**), n = 12 (6 cell donors infected with 2 viral isolates) for viral donor 1, n = 12 (6 cell donors infected with 2 viral isolates) for viral donor 2, and n = 30 (6 cell donors infected with 5 viral isolates) for viral donor 3. Statistics by Kruskal–Wallis test. *p < 0.05, **p < 0.01, ***p < 0.001.

In order to understand the entry properties of HIV derived from AMs, we isolated HIV *env* from lung and plasma samples. We sequenced HIV *env* RNA from AMs (27 isolates), BAL fluid (19 isolates), and plasma (21 isolates) from 3 HIV-infected ART-naive individuals (Supplementary Fig. 2). These sequences clustered by HIV-infected donor, but there was no compartmentalization when all AM-derived isolates were compared to all plasma-derived isolates. However, compartmentalization between AM and plasma derived sequences was observed in 2 of 3 donors (Supplementary Fig. 2). To test the ability of these AM- and plasma-derived Env proteins to allow infection of CD4+ T cells and MDMs, we cloned the *env* genes into an NL4-3 *env* deleted HIV backbone to generate replication-competent virus. We tested viruses containing Env from 8 AM and 9 plasma isolates selected to represent the sequence diversity found in the samples (arrows in Supplementary Fig. 2). Viruses containing all of the AM- and plasma-derived HIV Env isolates productively infected CD4+ T cells but did not infect MDMs (Fig. 2 and Supplementary Fig. 3). These data indicate that all AM- and plasma-derived HIV primary isolates found in our study were T-tropic.

**Figure 3:**
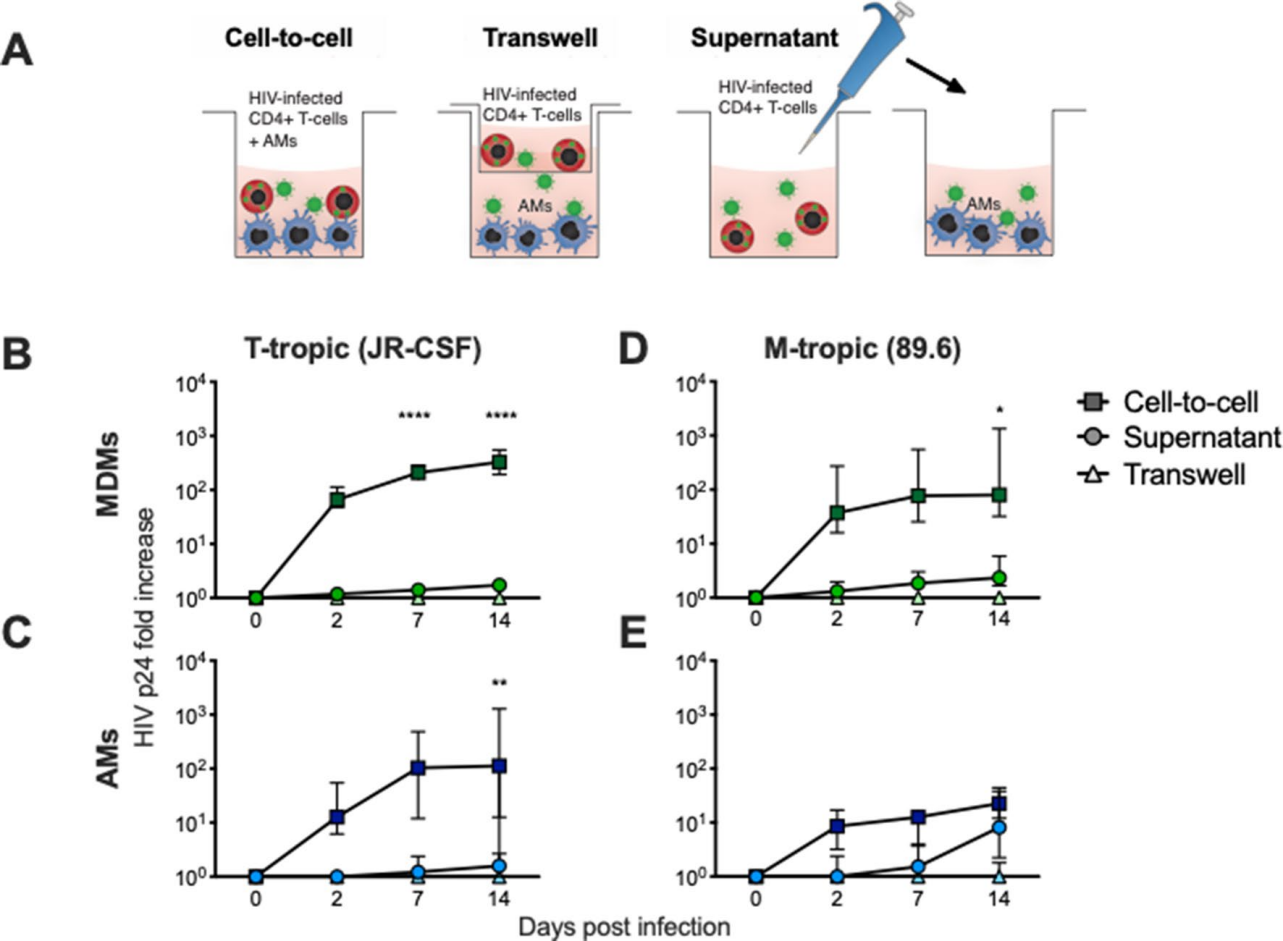
T-tropic HIV infection is more efficient through cell-to-cell than through cell-free transmission to MDMs and AMs. (**A**) MDMs were matured for 7 days or AMs were adhered for 1 h. CD4+ T cells were activated with PHA for 3 days, infected with HIV JR-CSF or 89.6 at a MOI of 0.2 overnight, washed, and cultured for 4 days, when approximately 10% of T cells were HIV Gag+ by flow cytometry. After the T cells were centrifuged, the supernatant from CD4+ T cells was used for the SN condition. The pellet of CD4+ T cells was resuspended and added directly (CTC) or across a transwell (TW) for 3 h and washed, using a ratio of 1:1 T cell:macrophage. (**B**–**E**) Supernatants from (**B**, **D** ) MDMs or (**C**, **E**) AMs were collected and HIV p24 was measured by ELISA. *p* value shows the value for cell-to-cell vs. supernatant conditions at the given timepoint using a two-way ANOVA with Tukey’s multiple comparisons test; n = 6 for MDMs and AMs. *p < 0.05, **p < 0.01, ****p < 0.0001.

### T-tropic HIV efficiently infects AMs through contact with infected CD4+ T cells

Previous work has shown increased efficiency of M-tropic HIV entry into MDMs through uptake of or fusion with infected T cells^44,45^. However, whether this also occurs with T-tropic HIV, the primary virus during acute and chronic infection, and its relevance to primary AM infection remains unknown. To test T-tropic and M-tropic HIV entry into AMs via interaction with HIV-infected T cells, we infected PHA-activated CD4+ T cells with the CCR5-using T-tropic HIV strain JR-CSF. We then cultured HIV from these autologous infected CD4+ T cells with MDMs or AMs in the following conditions: (1) infected CD4+ T cells added directly to the macrophages (“cell-to-cell” or “CTC”); (2) infected CD4+ T cells separated from the macrophages by a transwell (“TW”); and (3) infected CD4+ T cell culture supernatant added to the macrophages (“SN”) (Fig. 3A). The cells or supernatant were added for 3 h before washing the macrophages. In MDMs and AMs, T-tropic HIV replication was significantly increased by contact with infected CD4+ T cells compared to the SN or TW conditions (Fig. 3B, C). Other reports have suggested that contact with T cells enhances M-tropic infection rates of MDMs^44,45^, but this has not been shown in AMs. To test this, we repeated the experiment with CD4+ T cells infected with the CCR5 and CXCR4-using M-tropic HIV strain 89.6. In MDMs, M-tropic infection was enhanced by contact with infected CD4+ T cells, similar to previous literature (Fig. 3D). However, in AMs, M-tropic HIV p24 levels were similar in the SN and CTC conditions by day 14 (Fig. 3E). In order to study whether productive HIV infection occurs in the T cell-macrophage co-cultures, we pre-treated the macrophages and T cells with the non-nucleoside reverse transcriptase inhibitor (NNRTI) efavirenz (EFV). We found that EFV pre-treatment inhibited T-tropic HIV replication by day 14 in the CTC condition in both MDMs and AMs (Supplementary Fig. 4A, B). These data demonstrate that T-tropic HIV infects MDM and AM cultures more efficiently through contact with an infected CD4+ T cell intermediate, and that co-culture of T-tropic HIV-infected CD4+ T cells with MDMs or AMs leads to productive infection.

**Figure 4:**
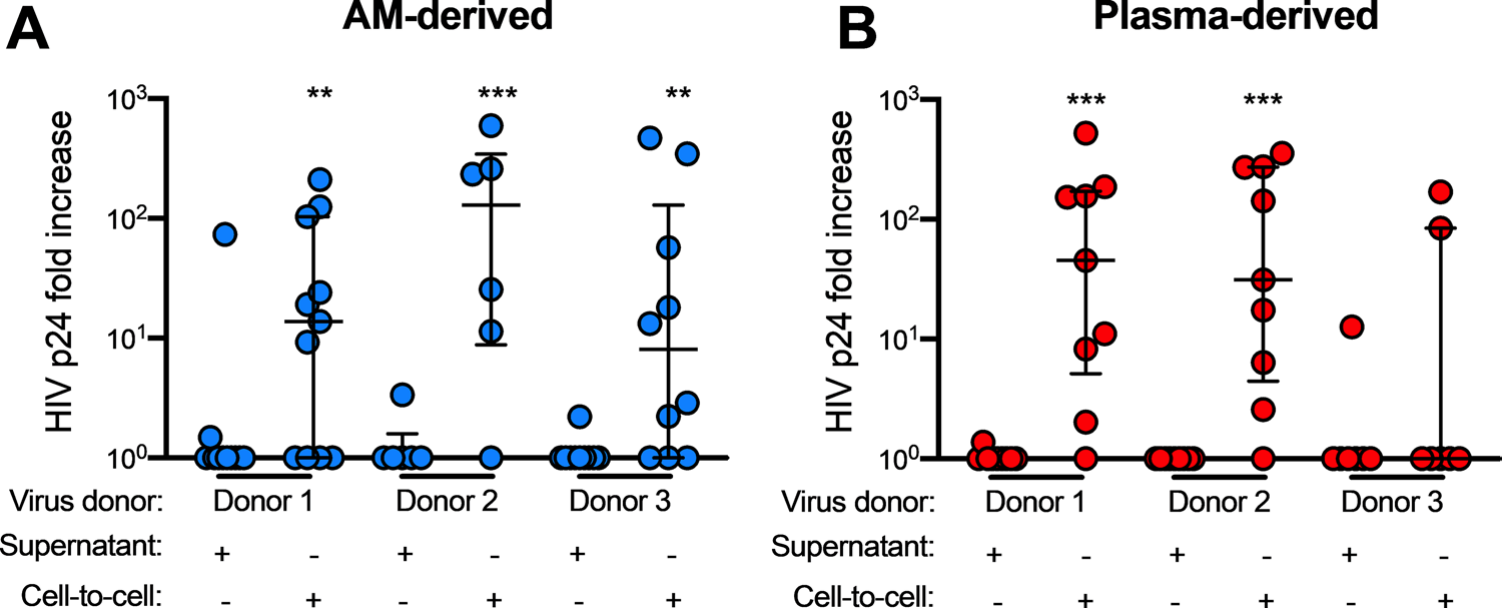
Viral constructs made with patient-derived HIV *env* from AMs and plasma productively infect MDMs more efficiently through T cell contact. Experimental conditions were as in Fig. 3 with the HIV constructs described in Fig. 2. Cells were infected with AM-derived (**A**) or plasma-derived (**B**) viral isolates from viral Donors 1, 2 or 3. CD4+ T cells and MDMs were isolated from the same blood donors. In (**A**), n = 11 cell donors for viral donor 1, n = 6 cell donors for viral donor 2, and n = 10 cell donors for viral donor 3; in (**B**), n = 9 cell donors for viral donor 1, n = 9 cell donors for viral donor 2, and n = 7 cell donors for viral donor 3. *p* value from Kruskal–Wallis test. Statistics compare the fold change at the final timepoint to day 1. **p < 0.01, ***p < 0.001.

### AM-derived HIV can productively infect MDMs more efficiently when there is contact with infected CD4+ T cells

Having established that infection of AMs and MDMs with a laboratory strain of T-tropic HIV can be enhanced by contact with an infected CD4+ T cell, we tested whether infection with primary patient-derived AM- or plasma-derived HIV isolates could also be enhanced in macrophages through this same route. Using the experimental setup described above, we demonstrate that both AM-derived and plasma-derived primary isolates did not productively infect MDMs in the SN condition (Fig. 4 and Supplementary Fig. 5), consistent with our observations of the T-tropic virus JR-CSF. On rare occasions, there was detectable p24 production in the cell-free virus (SN) condition. However, for all 3 AM-derived and 2 out of the 3 plasma-derived Envcontaining viruses, infection of MDMs was observed in the CTC condition (Fig. 4 and Supplementary Fig. 5). These data indicate that primary HIV isolates made with Env derived from AMs infect MDMs inefficiently on their own, but can infect MDMs more efficiently via contact with an infected CD4+ T cell intermediate.

**Figure 5:**
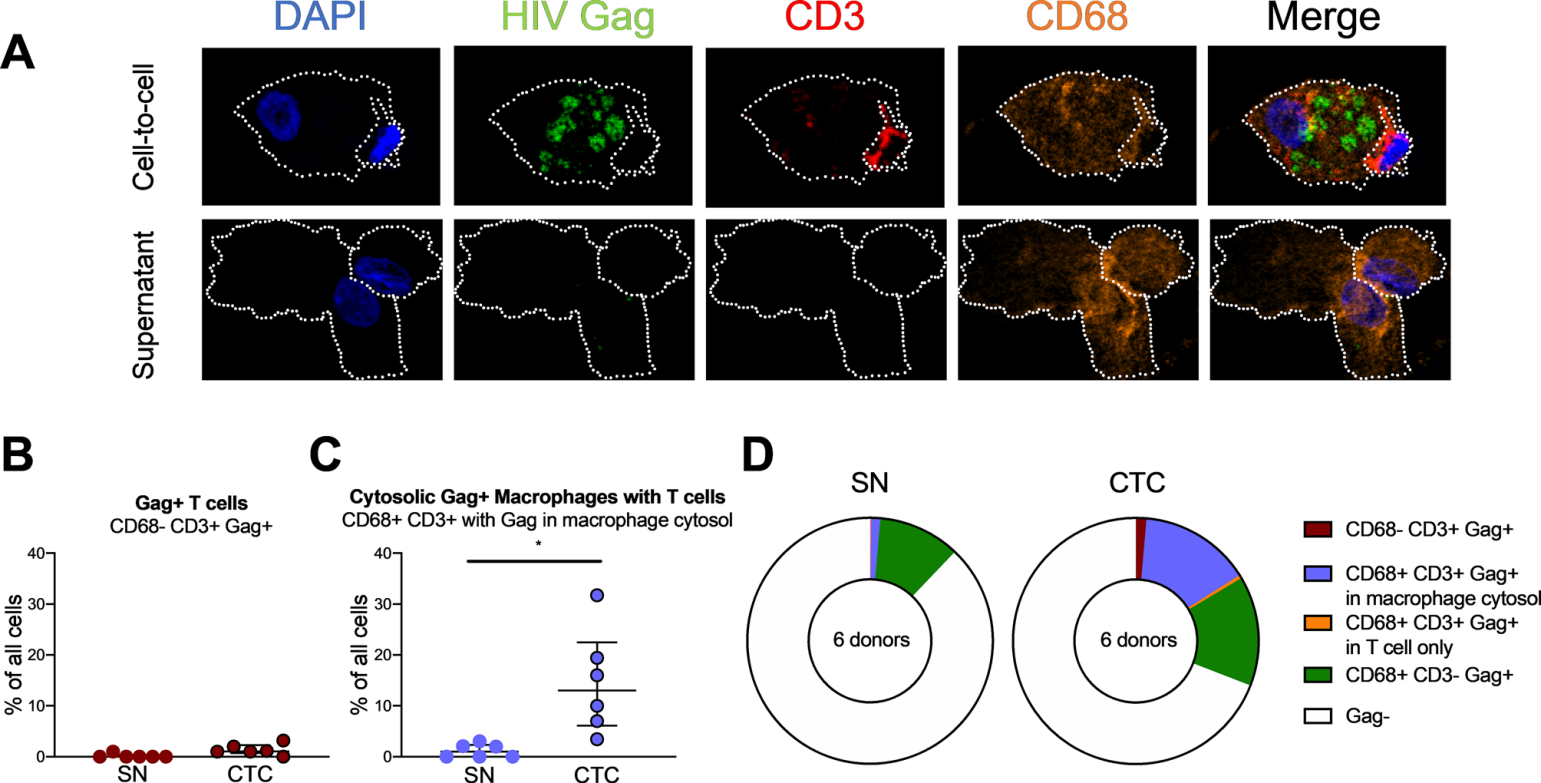
T-tropic HIV-infected T cell contact with AMs leads to HIV Gag staining in the AM cytosol. (**A**) AMs were co-cultured with HIV JR-CSF-infected autologous CD4+ T cells (cell-to-cell, CTC) or T cell supernatant (SN) for 3 h, washed, and cultured for an additional 14 days. Staining was performed with DAPI for cell nuclei, HIV Gag, CD3 for T cells, and CD68 for macrophages, and images were acquired by immunofluorescent confocal microscopy. White dotted outlines show the AM or T cell membrane. (**B**–**D**) % Gag+ cells out of all nucleated cells were quantified. (**B**) Gag + CD4+ T cells not associated with AMs (Gag+ T cells) were rare in both the SN and CTC conditions. (**C**) Cytosolic gag staining was increased in AMs associated with CD3+ T cells in the CTC condition compared to the SN condition. (**D**) Pie charts of all of the staining categories in SN and CTC conditions. Gag staining was rarely observed in solitary CD3+ T cells. Each category was compared between SN and CTC by the Friedman test with Dunn’s multiple comparison’s test. 100 cells per donor were quantified; n = 6 donors. *p < 0.05.

### T cell-mediated infection of macrophages is mainly associated with CD4+ T cell contact

To further characterize the cellular location of HIV in infected macrophages, we imaged T cell-macrophage co-cultures using immunofluorescent confocal microscopy. As before, autologous CD4+ T cells were infected with T-tropic HIV, and T cell supernatant (SN) or HIV infected CD4+ T cells (CTC) were added to AMs. The samples were stained with anti-CD3 (T cells), anti-CD68 (macrophages) and anti-HIV Gag, and imaged after 14 days (Fig. 5A). Staining was quantified to localize HIV Gag. As expected, we detected Gag staining within AMs primarily in the CTC but not in the SN condition (Fig. 5A and Supplementary Fig. 6A). The CD3 staining was limited to a discrete intracellular area, suggestive of an endocytosed CD3+ T cell. Gag+ CD3+ T cells that were not associated with AMs were rare, with a median of 0% and 1.07% of all cells for JR-CSF SN and JR-CSF CTC, respectively (Fig. 5B), indicating that the increase in HIV release in co-culture experiments was likely not due to infection of T cells alone. Interestingly, the majority of the HIV-infected AMs were closely associated with T cells (CD68+ CD3+) and had HIV Gag staining within the macrophage cytosol (“CD68+ CD3+ with Gag in macrophage cytosol”; Fig. 5C and D). Some of these T cells were fully internalized into the macrophage, but other T cells were in close contact with the AM, suggesting that T cell internalization was not required for HIV Gag staining in the AM cytosol. HIV Gag was rarely observed only within the cytosol of T cells internalized by AMs without HIV Gag staining also in the macrophage cytosol (“CD68+ CD3+ Gag+ in T cell only”; Fig. 5D and Supplementary Fig. 6B). Both the SN and CTC conditions had a similar percentage of Gag+ AMs with no T cell staining (Fig. 5D, Supplementary Fig. 6C), which may be the result of archiving of HIV in endosomal compartments^51^. The percentage of Gag+ AMs with no T cell staining was the same on day 0 after 3 h of incubation and on day 14, supporting the conclusion that this staining in cell-free infection is due to phagocytosis or archiving (data not shown). These findings show that T-tropic HIV can be found in the AM cytosol after co-culture with infected T cells, suggesting that HIV is transmitted from an infected CD4+ T cell to the AM cytosol, and results in productive infection of AMs.

## Discussion

While HIV nucleic acid has been detected in AMs during early stages of HIV infection, circulating HIV at this stage is primarily T-tropic and enters macrophages inefficiently in vitro. The tropism of HIV from AMs and the mechanism of HIV entry into AMs are both unknown, and may have a significant impact on the burden of pulmonary disease observed in those living with HIV. We studied AMs obtained by bronchoscopy in a cohort of ART-naive HIV-infected and uninfected individuals in Cape Town, South Africa and demonstrate that HIV RNA and DNA can be detected within AMs. We show that AM-derived HIV isolates are T-tropic and efficiently infect T cells but not macrophages; however, these isolates can productively infect macrophages through contact with infected CD4+ T cells. Our findings indicate that CD4+ T cell-dependent infection of AMs is a route of infection that may explain the presence of T-tropic HIV in AMs despite inefficient cell-free infection. This route of AM infection may be an important contributor to the burden of pulmonary disease observed in those living with HIV infection.

A few characteristics of the study population are notable, including sensitization to *Mycobacterium tuberculosis* and frequency of detection of HIV nucleic acids. The volunteers in the study were all sensitized to *Mycobacterium tuberculosis*, which could potentially increase the ability of AMs to be infected by HIV, including increased replication of HIV at sites of TB infection^52–55^. This reflects the high rates of latent tuberculosis infection seen in this population. However, the detection frequency of HIV Gag RNA and DNA were consistent with other studies, including those performed in non-TB sensitized populations. We observed HIV Gag RNA in AMs from 36.4% of participants and Gag DNA in AMs from 45.5% of participants, which is consistent with studies using qPCR to detect HIV nucleic acids in AMs^18,20,21,40,46–50^.

To better understand the phenotype of HIV derived from AMs, we generated replication-competent strains of HIV using *env* isolated from individuals with untreated HIV infection. We found that 8 separate AM-derived viruses and 9 plasma-derived viruses, selected to maximize sequence diversity, all had a T-tropic phenotype. This suggests that T-tropic HIV is infecting AMs in vivo despite inefficient infection of macrophages by these isolates in vitro. We used PCR-based methods to amplify virus, which could result in HIV recombination within the samples. However, the consistency of the entry phenotype across separate AM-derived Env constructs suggests that the tropism of macrophage-derived Env is more broadly applicable. In addition, the adherence method of isolating AMs from BAL, while resulting in 99–100% macrophage purity by RapidDiff stain, does not allow us to rule out a contribution of residual infected T cells to the HIV we detected and isolated. This method has been used in recent prior literature measuring HIV in AMs^22,56^ and led to minimal T cell inclusion in our co-culture studies, but could have resulted in leftover HIV-infected CD4+ T cells. A third caveat is that cell-free virus occasionally replicated over time in the cell-free conditions, although more rarely than with cell-to-cell contact. This phenomenon suggests that cell-free infection of MDMs by this T-tropic virus is possible but is less efficient than cell-to-cell infection. M-tropic HIV strains have 30-fold more efficient entry into macrophages than T-tropic strains do^23^, which is consistent with the idea that T-tropic strains occasionally enter MDMs.

Our data suggests that the p24 increase in AM-T cell co-cultures is due to productive infection of AMs. We cannot rule out new infection of CD4+ T cells that remained in the co-cultures after three rounds of washing. Treatment of these co-cultures with the reverse transcriptase inhibitor efavirenz inhibited viral replication. This shows that new viral production originated from cells infected after co-culture of CD4+ T cells and macrophages, and did not come from viral release from residual infected cells. It is possible that the newly infected cells are CD4+ T cells. However, in our microscopy staining, CD4+ T cells were at low prevalence (Fig. 5B), and most of the HIV Gag staining was localized to macrophages (Fig. 5D). Our work indicates that cell-to-cell infection of macrophages may be an important mechanism of T-tropic viral entry into AMs in the acute and chronic stages of infection.

While dendritic cells, alveolar epithelial cells, CD8+ T cells, NK cells and neutrophils may impact HIV infection dynamics in the alveolar space, either directly by acting as targets for infection or indirectly through cytokine secretion^57^, the main cells infected with HIV in the lung in vivo are CD4+ T cells and alveolar macrophages^58,59^.

A number of possibilities may explain the mechanism of the observed enhancement. The formation of an immunological and/or virological synapse, which involves the clustering of immune receptors and HIV virions at the interface between an AM and CD4+ T cell, may help overcome the low density of HIV entry receptors on AMs^44,60,61^. Fusion of the infected CD4+ T cell with the macrophage is another proposed mechanism of HIV infection of macrophages^45^, although we did not observe multinucleated giant cells or macrophages with surface CD3 expression, suggesting that this may not be occurring in our system with high frequency. It is also possible that autologous CD4+ T cells may activate AMs and increase HIV transcription^62^, as has been shown in dendritic cells^63^. In the CTC condition, we observed AMs with cytosolic Gag staining with T cells closely apposed to the membrane or internalized. This implies that AM phagocytosis of T cells occurs but may not be required for transmission of HIV Gag to the AM. Future work is needed to clarify this mechanism and understand its implications for HIV compartmentalization within AMs.

Infected AMs have been observed both in ART-naïve and ART-treated individuals with HIV^9,16–22,64,65^. ART may be able to inhibit HIV infection of new AMs in vivo as well as in vitro, as suggested by the ability of efavirenz to inhibit productive MDM infection (Supplementary Fig. 3A). However, HIV-infected macrophages are known to persist for long periods because they are more resistant to the cytopathic effects of HIV^35^, the cytotoxic activity of CD8+ T cells^66^, and apoptosis^67–69^. Infected AMs may persist during ART treatment and may contribute to the dysfunction^22,69^ either directly or through indirect effects of HIV components or other factors on bystander AMs^70^. Persistent AM dysfunction may contribute to the increased rate of lung inflammation and infectious disease seen in people with HIV on ART^71–74^. An understanding of the mechanism behind AM infection may help efforts to address persistent sources of inflammation in the lung in people with HIV on ART.

ART reverses some but not all of the functional phagocytic defects in alveolar macrophages, which may be due to a differential impact of ART on infected and bystander AMs^75^. Direct infection of macrophages can activate immune pathways including type I interferons that affect bystander function^76,77^ and chemokines which recruit other immune cells^66,78^. A number of papers have explored mechanisms of macrophage functional impairment in people with HIV on ART that are not dependent on direct infection, which include gp120-induced inhibition of apoptosis^69^, nef-induced inhibition of phagocytosis^8,79^ and downregulation of CD36^80^, as well as post-translational modification of Fc receptors^81^ and changes in reactive oxygen species generation^82^. Notably, HIV proteins including gp120 and nef persist in bronchoalveolar lavage fluid in people with HIV on ART^69,83^. Finally, data from ART-naïve macaques shows that only about 1 in 20,000–100,000 AMs is productively infected with SIV^84^, so HIV-infected AMs likely only make up a small minority of AMs in the lung in ART-naïve or treated individuals. The mechanisms of HIV-mediated AM impairment, even in the absence of ART, are most likely attributable to an indirect effect of either HIV or soluble factors produced by infected AMs on uninfected AMs.

The finding that HIV isolated from AMs is T-tropic has important implications for our understanding of the HIV reservoir. The vast majority of HIV strains, including transmitted/founder strains, are T-tropic^25,33^. If T-tropic HIV is able to infect AMs, the majority of people with HIV have the potential to have infected AMs, as well as infected macrophages in other tissues. This has implications for macrophages as a reservoir for HIV. It also suggests that tissue macrophages may need to be targeted in cure strategies.

The potential for AMs to be productively infected by contact with an infected CD4+ T cell intermediate has several important implications for our understanding of HIV pathogenesis. Productive HIV infection has been shown to have many effects on macrophage function, including the generation of defects in phagocytosis, proteolysis, and cytokine production^9^. Additionally, infected macrophages may efficiently transmit HIV to other CD4+ T cells and macrophages, as has been shown for M-tropic HIV, through efferocytosis of infected macrophages or cell-to-cell contact^61,85^. HIV-infected macrophages are resistant to cell death and in non-human primates can harbor replication-competent SIV after ART^65^, leading to a cellular reservoir that may play a role in the failure of current attempts at HIV cure. Finally, HIV infection in macrophages via contact with T cells may play a role in the chronic immune activation that is seen with HIV infection. Multiple studies have associated macrophage-derived products with increased morbidity and mortality during HIV infection, including those on ART. These include IL-6, soluble CD14 (sCD14), soluble tumor necrosis factor receptor 1 (sTNFR1), sTNFR2, and indoleamine 2,3-dioxygenase (IDO) activity^86–89^. Therefore, AMs infected with T-tropic HIV via T cell contact may lead to multiple immune defects which may contribute to an increased incidence of lung disease and promotion of HIV disease progression in people living with HIV.

## Methods

### Human participants

Bronchoscopies were performed in a cohort of participants with HIV infection and uninfected control individuals residing in Cape Town, South Africa, as previously described in^90^. All participants in the cohort were sensitized to *Mycobacterium tuberculosis* as defined by positive results of an interferon γ release assay (IGRA; Quantiferon, Cellestis), without active TB as defined by absence of signs or symptoms of active TB, lack of clinical findings by chest X-ray, and a negative BAL TB culture. All volunteers who had previously been diagnosed with or treated for TB were excluded. The HIV-infected participants in the Cape Town cohort were asymptomatic and not using antiretroviral therapy. All HIV + IGRA + volunteers were eligible for INH prophylaxis according to South African National guidelines, and were offered isoniazid prophylactic treatment for 6 months, which was the indication at the time. All samples were taken prior to INH treatment.

For in vitro experiments, BAL was also collected from a cohort of HIV-uninfected individuals, recruited from outpatient clinics at local Boston hospitals, following institutional review board approval (IRB protocol # 2013P000063) and written informed consent. For HIV infection experiments involving monocyte derived macrophages (MDMs), cells were isolated from buffy coats of anonymous healthy blood donors obtained from the Massachusetts General Hospital (MGH) blood donor center (Boston, MA) under protocol # 2005P001218.

### Bronchoalveolar lavage (BAL)

Bronchoscopies were performed under conscious sedation via standard technique^15,90^. For each BAL in the Cape Town cohort, samples were collected from the right middle lobe by washing with 160 ml of normal saline. After adherence, approximately 10,000 cells were centrifuged onto a coated microscope slide using a Cytospin Centrifuge (Shandon 3.0). The slide was then allowed to dry, dipped repeatedly in fixative and differentially stained by dipping the slide consecutively in two contrasting dyes (RapidDiff Kit, Clinical Sciences Diagnostics). The differential stain allows characterization of lymphocytes, macrophages and neutrophils, based on morphology. The slide was then viewed under immersion oil magnification using a light microscope and a total of 200 cells were counted. The BAL samples consisted of a median of 96% and 93% macrophages prior to adherence, in the HIV+ and HIV- BAL samples, respectively. This was enriched further by adherence for 20 min in order to eliminate the non-adherent cell fraction.

For the Boston cohort, samples were collected from the lingula and the right middle lobe by washing 120 ml of normal saline in each segment. Alveolar macrophages (AMs) from bronchoscopy participants were isolated by 20–60 min of adherence. AMs for in vitro infection were washed 3 times with PBS and cultured in RPMI with 10% (v/v) FCS. BAL CD4+ T cells were obtained at bronchoscopy of ART-naive uninfected participants using a Human CD4+ T cell Enrichment kit (StemCell EasySep) on non-adherent BAL cells.

### Cell isolation from blood

Peripheral blood from the bronchoscopy study participants recruited in Boston was obtained at least 7 days before the bronchoscopy and PBMCs were processed and cryopreserved. Ficoll gradients were used to isolate peripheral blood mononuclear cells (PBMCs). Monocytes and CD4+ T cells were then isolated by CD14+ positive selection (Miltenyi) and CD4+ T cell negative selection (StemCell EasySep), respectively. MDMs were obtained by maturing monocytes in RPMI with 10% GemCell US Origin Human Serum AB (GemBio) for 7 days.

### Measurement of HIV nucleic acids

For measurement of HIV RNA and DNA, AMs were lysed in RLT Plus lysis buffer (QIAGEN) with 1% β-mercaptoethanol (Sigma). The lysate was run through QIAShredder columns (QIAGEN), and isolated with AllPrep Micro DNA/RNA kits (QIAGEN). HIV Gag primers were used to obtain the number of copies of HIV RNA and DNA, and CCR5 primers were used to determine the number of cells per sample using TaqMan qRT-PCR (Applied Biosystems)^91^. Standard curves were prepared using preamplification of a purified restriction digested HxB2 plasmid for HIV gag, kindly provided by Todd Allen and Karen Power, and by preamplification of a CCR5 plasmid^91^.

### HIV production

Cells were infected with CCR5-utilizing T-tropic (JR-CSF) or CCR5/CXCR4-utilizing M-tropic (89.6) HIV constructs. JR-CSF was obtained through the NIH AIDS Reagent Program, Division of AIDS, NIAID, NIH: HIV JR-CSF Infectious Molecular Clone (pYK-JRCSF) (Cat# 2708) from Dr. Irvin SY Chen and Dr. Yoshio Koyanagi^92–94^; 89.6 was obtained as above as the HIV 89.6 Infectious Molecular Clone (p89.6) from Ronald G. Collman, MD (cat# 3552)^95–97^. For quantification of entry, the JR-CSF and 89.6 viruses were produced with the BLaM-Vpr reporter plasmid^98,^ kindly provided by Blandine Monel. Production of HIV was performed by transfecting viral plasmids with Hilymax (Dojindo) into HEK293T cells (ATCC), changing media after 12 h, harvesting cell culture supernatant after 48 h, and concentrating 50 times with PEG-it (System Biosciences). Supernatants were aliquoted and stored at  − 80 °C until further usage. Titers of HIV infectious particles/ml were determined by infection of CD4, CCR5 and CXCR4-expressing GHOST cells (NIH AIDS Research and Reference Reagent Program)^99^.

### HIV entry assay

The HIV entry assay was performed by infecting 100,000 BAL CD4+ T cells, AMs or MDMs with 10^5^ infectious units of BLaM-Vpr HIV in a polypropylene FACS tube in a total volume of 100 μl after 1 h of pretreatment with DMSO or 40 μM maraviroc (MVC, Sigma). The cells were incubated at 37 °C for 12 h, then washed three times. Staining was performed by adding CCF2-AM along with Fixable Viability Dye eFluor 780 (eBioscience) to the cells and assaying for cleavage by flow cytometry^98^.

### HIV infection of CD4+ T cells and macrophages

For cell-free infection experiments, 2 × 10^4^ infectious units of HIV, or for mock infection, media, were added to 10^5^ cells (MDMs, T cells or AMs) in 100 μl total volume for 12 h at 37 °C, then washed three times with PBS and replaced with 200 μl media. Supernatant was collected for p24 measurement at days 1 (1 h after changing media), 2, 7, and 14. CD4 + T cell cultures had low viability at day 14, consistent with prior literature^100^, so samples were not collected at this point, and day 7 was the final timepoint. HIV p24 levels were measured by p24 ELISA (PerkinElmer).

### Co-culture of HIV infected CD4+ T cells and macrophages

For co-culture experiments with HIV-infected T cells, CD4+ T cells from matched donors were activated for 3 days with 2 ng/ml phytohemagglutinin (PHA-P) (ThermoFisher) and 10 ng/ml IL-2 (kindly provided by Alicja Trocha and the Ragon Institute Protein Core). 2 × 10^4^ infectious units of HIV were added to 10^5^ cells in each well and spin-infected by centrifugation at 800 ×g for 90 min at 4 °C; the cells were cultured for 12 h, washed and the media was replaced^15^. On day 4 after infection, autologous HIV- or mock-infected T cells were spun at 500 ×g for 3 min, the supernatant was collected, and the T cells were resuspended in fresh media. MDMs or AMs were cultured with one of three preparations for a 1:1 ratio of T cells to macrophages: supernatant collected from the CD4+ T cells (supernatant or “SN”), washed CD4+ T cells (cell-to-cell or “CTC”), or washed CD4+ T cells in the upper chamber of a 0.4 μm transwell (Transwell Costar) (transwell or “TW”). After incubation for 3 h at 37 °C, the cells were washed three times for 60 s with ice cold PBS with 10 mM EDTA, and media was replaced. Supernatant was collected for p24 measurement at days 0 (1 h after changing media), 2, 7 and 14 from the same wells with collection and replacement of 25% of the media volume. p24 levels were measured by ELISA (PerkinElmer). For experiments with AMs, autologous PBMCs were thawed 7 days before the bronchoscopy, and CD4+ T cells were isolated and activated for three days as above. In Fig. 4 and Supplementary Fig. 5, samples were only included in the final dataset if there was detectable HIV infection in the CD4+ T cells before they were added to the macrophages.

### HIV primary isolate sequencing and production of primary isolates

Viral RNA was extracted from 140 μl of plasma and BAL fluid using a QIAamp viral RNA minikit (QIAGEN), and cDNA copies of the vRNA were generated by reverse transcription using the SuperScript III protocol (Invitrogen). Nested PCR was performed for the *env* gene as described in^101^, and amplicons were cloned into a pHDM vector using the In-Fusion HD Cloning kit (Clontech) and sequenced on an Illumina MiSeq.

Based upon analysis of *env* sequence diversity, 22 isolates were selected to represent the diversity of sequences from the 3 donors, with at least one sequence from each clade (arrows in Supplementary Fig. 2). pNL4-3 Δ*Env* GFP was obtained through the NIH AIDS Reagent Program, Division of AIDS, NIAID, NIH: HIV NL4-3 Δ*env* EGFP Reporter Vector from Drs. Haili Zhang, Yan Zhou, and Robert Siliciano (cat# 11100)^102^. The amplicons were PCR amplified from the pHDM vectors and cloned into the pNL4-3 vector. HIV stocks were produced, frozen and titered on GHOST cells as above. Experiments in Fig. 2 and Supplementary Fig. 3 used 2 AM-derived and 2 plasma-derived viral isolates from donor 1, 3 AM derived and 2 plasma-derived viral isolates from donor 2, and 3 AM derived and 5 plasma-derived viral isolates from donor 3, which are indicated by arrows with and without a black border in Supplementary Fig. 2. Experiments in Figs. 4 and 5 used one viral isolate from each viral donor and compartment, which are indicated by arrows without a black border.

### Microscopy

Poly-L-lysine coated coverslips were used to culture MDMs and AMs with infected CD4+ T cells or supernatant from infected CD4+ T cells under SN or CTC conditions as described above. Cells were fixed with 4% paraformaldehyde, permeabilized with 0.1% saponin, blocked with 1% BSA, and stained with antibodies against HIV Gag (conjugated to FITC, Beckman Coulter), CD3 (rabbit polyclonal, Dako), and CD68 (KP1, BioCare Medical), then Alexa Fluor 568 Goat anti-Rabbit and Alexa Fluor 647 Goat anti-Mouse (Invitrogen), then DAPI and mounted with ProLong Diamond mounting media (Thermo Fisher). Slides were scanned with a TissueFAXS SL Q spinning disc Confocal microscope (TissueGnostics USA) using a Zeiss Plan-apochromatic 63 × 1.4NA oil immersion objective. Image analysis involved counting 100 cells from each slide on a single Z-stack image and classifying each cell as CD68± , CD3± , and Gag±, and if Gag+ , whether it was present in CD68+ and/or CD3+ areas of the cell.

### Quantification and statistical analysis

Statistical details of experiments can be found in each figure legend. Nonparametric tests were used to compare medians between groups unless noted otherwise. The Mann–Whitney test was used for 2 groups and the Kruskal–Wallis test followed by Dunn’s multiple comparison post-test was used for > 2 groups. Wilcoxon signed rank was used to compare continuous data between two time points. Spearman’s correlation coefficients were used to examine associations between variables. Differences were considered significant at *p* < 0.05. For figures marked “fold increase”, the value at the final timepoint was compared to the initial timepoint; rare values below 1 were normalized to the value of 1. Prism 8 (Graphpad) was used for all analyses^15^.

### Ethical statement

All participants provided prior written informed consent. The study was conducted in accordance with the Declaration of Helsinki. The work involving bronchoalveolar lavage samples from Cape Town, South Africa was approved by the Research Ethics Committees of the University of Cape Town (REF158/2010) and Stellenbosch University (N10/08/275). All work involving material from human subjects performed in Boston, MA, USA was approved by the Institutional Review Board (IRB) at Massachusetts General Hospital (MGH). The work involving bronchoalveolar lavage samples from Boston, USA was approved by the Partners Human Research Committee (2013P002436), and the collection of blood samples for PBMC isolation was approved by the Partners Human Research Committee (2005P001218).

## Supplementary Information

Supplementary Information.

Supplementary Figure 1.

Supplementary Figure 2.

Supplementary Figure 3.

Supplementary Figure 4.

Supplementary Figure 5.

Supplementary Figure 6.
