# Supplementary material for: T cell-tropic HIV efficiently infects alveolar macrophages through contact with infected CD4+ T cells
Source: Sci Rep. 2021 Feb 16;11:3890. doi: 10.1038/s41598-021-82066-x (PMC7886866; doi:10.1038/s41598-021-82066-x)
Supplement: Supplementary file 1 — Supplementary Information. [file 41598_2021_82066_MOESM1_ESM.docx]

**T cell-tropic HIV efficiently infects alveolar macrophages through contact with infected CD4+ T cells**

Abigail E. Schiff^1,2^, Alice H. Linder^1^, Shillah N. Luhembo^1^, Stephanie Banning^3^, Martin J. Deymier^1^, Thomas J. Diefenbach^1^, Amy K. Dickey^4^, Athe M. Tsibris^2,3^, Alejandro B. Balazs^1^, Josalyn L. Cho^4,#a^, Benjamin D. Medoff^4^, Gerhard Walzl^5^, Robert J. Wilkinson^6,7,8^, Wendy A. Burgers^6,9^, Björn Corleis^1,#b,#c,*^, and Douglas S. Kwon^1,2,10,#c,*^

^1^ Ragon Institute of MGH, MIT, and Harvard, Massachusetts General Hospital, Harvard Medical School, Cambridge, MA, United States of America

^2^ Harvard Medical School, Boston, MA, United States of America

^3^ Division of Infectious Diseases, Brigham and Women’s Hospital, Harvard Medical School, Boston, MA, United States of America

^4^ Division of Pulmonary and Critical Care Medicine, Massachusetts General Hospital, Boston, MA United States of America

^5^ DST-NRF Center of Excellence for Biomedical Tuberculosis Research, South African Medical Research Council Centre for Tuberculosis Research, Division of Molecular Biology and Human Genetics, Faculty of Medicine and Health Sciences, Stellenbosch University, Cape Town, South Africa

^6^ Wellcome Center for Infectious Diseases Research in Africa and Institute of Infectious Disease and Molecular Medicine, University of Cape Town, Observatory 7925, Republic of South Africa

^7^ Department of Infectious Disease, Imperial College London, W12 ONN, United Kingdom

^8^ The Francis Crick Institute, 1 Midland Road, London, NW1 AT, United Kingdom

^9^ Division of Medical Virology, Department of Pathology, University of Cape Town, Cape Town, Republic of South Africa

^10^ Division of Infectious Diseases, Massachusetts General Hospital, Boston, MA, United States of America

^#a^ Division of Pulmonary, Critical Care and Occupational Medicine, University of Iowa, Iowa City, IA, United States of America

^#b^ Institute of Immunology, Friedrich-Loeffler-Institute, Federal Research Institute for Animal Health, Greifswald, Isle of Riems, Germany

^#c^ Contributed equally to this work

* Co-corresponding author

E-mail: dkwon@mgh.harvard.edu (DSK); Bjoern.Corleis@fli.de (BC)

**Supplementary methods**

**Measurement of HIV nucleic acids**

For quantification of HIV Gag RNA, the HIV Gag primers SK145 (forward): AGTGGGGGGACATCAAGCAGCCATGCAAAT and SK431 (reverse): TGCTATGTCACTTCCCCTTGGTTCTCT (*1*) were used. For quantification of HIV Gag DNA, the primers HIV-FOR (forward): TACTGACGCTCTCGCACC, HIV-REV (reverse): TCTCGACGCAGGACTCG, and Probe: 5’ FAM-CTCTCTCCTTCTAGCCTC-MGB 3’ (Life Technologies) were used. For quantification of cellular DNA, the CCR5 primers CCR5 F (forward): ATGATTCCTGGGAGAGACGC, CCR5 R (reverse): AGCCAGGACGGTCACCTT, and Probe: 5’ FAM-AACACAGCCACCACCCAAGTGATCA-BHQ (Sigma) were used (*2*).

**Flow cytometry**

For analysis of T cell and macrophage HIV receptor expression, cell pellets were blocked with Human TruStain FcX Fc Receptor Blocking Solution (BioLegend), and stained with antibodies against CD3 (UCHT1, BD Biosciences), CD4 (RPA-T4, eBioscience), CCR5 (2D7, BD Biosciences), CXCR4 (12G5, R&D Systems), CD163 (GHI/61, BD Biosciences), and CD14 (15-2, BD Biosciences), along with blue viability dye (Invitrogen fixable viability dye), then fixed and run on a 4 laser LSR Fortessa flow cytometer (BD Biosciences). Flow data were analyzed with FlowJo (TreeStar) using fluorescence minus one controls to set gates on viable single cells.

**Viral isolation, sequencing and cloning**

The primers used in these reactions were 5′-GCAATAATTGTGTGGTCCATAGTACTCATAGAATATAGGA and 3′-CCCTATCTGTTGCTGGCTCAGCTCGTC for the first round and 5′-AAAATAGATAGGTTAATTGATAAAATAAGAGAGAGAGCAGAAGACAG and 3′-TCATTCTTTCCCTTACAGTAGACCATCCAGGC for the second round, targeting the *env* gene of HIV JR-CSF strain (*3*). The second-round PCR product was purified by agarose gel electrophoresis and gel extraction (Bioland), and the product was cloned by homologous recombination into a pHDM vector using the In-Fusion HD Cloning kit (Clontech). The product was transformed into DH5α competent cells, and positive clones were selected for sequencing. Envelope sequences were analyzed using the Geneious package ([www.geneious.com](http://www.geneious.com)). After sequencing, selected isolates were cloned into a replication-competent HIV backbone. Two isolates were selected from Donor 1 AMs, two from Donor 1 plasma, three from Donor 2 AMs, three from Donor 2 cell-free BAL fluid, two from Donor 2 plasma, three from Donor 3 AMs, two from Donor 3 BAL fluid, and five from Donor 3 plasma. To amplify the isolates from the pHDM backbone, the primers Env_pNL43 (forward) 5’-GCCATAATAAGAATTCATGAGAGTGACGGGGA-3’ and Env_pNL43 (reverse) 5’-TAAGTGCTAAGGATCCTTATAGCAAAGCTGCTTCAA-3’ were used with the CloneAmp HiFi PCR Premix (Takara Bio), and then cloned into the pNL4-3 vector with the In-Fusion HD Cloning kit (Clontech). Plasmids were screened with restriction digestion and sequenced at the MGH sequencing core.

**Co-culture of HIV infected CD4+ T cells and macrophages**

For experiments using Efavirenz (EFV), macrophages and T cells were pre-treated with DMSO or 100 nM EFV for 1 hour at 37°C. The experiments shown in Fig 4 were performed at 10^5^ macrophages/well in 96-well plates, and 10^5^ CD4+ T cells were added per well; the experiments shown in Figs 3 and 5 and Supplementary Fig 4 were performed at 5 x 10^5^ macrophages/well in 24 well plates, with 5 x 10^5^ CD4+ T cells added per well, in order to perform microscopy on the samples.

**Supplementary Tables and Figure Legends**

**Supplementary Table 1. Patient clinical characteristics in the Cape Town cohort.**

| **PID** | **HIV status** | **Age** | **Gender** | **CD4+ T cell count (cells/μl)** | **Plasma viral load** | **BAL viral load/ml ELF** | **Purity of adhered AMs under cytospin (%)** |
| --- | --- | --- | --- | --- | --- | --- | --- |
| 1032 | *‾* | 21 | F | ND | NA | NA | 100 |
| 1058 | *‾* | 35 | F | 866 | NA | NA | 100 |
| 1011 | *‾* | 27 | M | 801 | NA | NA | 99 |
| 1140 | *‾* | 28 | M | 423 | NA | NA | ND |
| 1014 | *‾* | 25 | F | 1159 | NA | NA | 99 |
| 1023 | *‾* | 18 | F | 1120 | NA | NA | ND |
| 1007 | *‾* | 18 | F | 1319 | NA | NA | 99 |
| 1066 | *‾* | 38 | F | 621 | NA | NA | 100 |
| 1060 | *‾* | 24 | F | 902 | NA | NA | 100 |
| 1024 | *‾* | 24 | M | 939 | NA | NA | 99 |
| 1031 | *‾* | 19 | F | 1169 | NA | NA | 100 |
| 1047 | *‾* | 26 | M | 680 | NA | NA | 100 |
| 1052 | *‾* | 26 | F | 1412 | NA | NA | 100 |
| 1057 | *‾* | 21 | M | 871 | NA | NA | 100 |
| 1076 | **+** | 26 | F | 543 | 908 | LDL | 99 |
| 1152 | **+** | 31 | F | 571 | 9697 | 17459 | 99 |
| 1151 | **+** | 27 | F | 749 | 40 | LDL | 99 |
| 1134 | + | 37 | F | 599 | 6383 | 8449 | ND |
| 1137 | **+** | 27 | F | 560 | 18797 | 60444 | ND |
| 1005 | **+** | 52 | F | 604 | 94 | LDL | 100 |
| 1131 | **+** | 32 | M | 785 | 331 | 2068 | 98 |
| 1133 | **+** | 30 | F | 511 | 40 | LDL | 98 |
| 1080 | **+** | 52 | F | 774 | 14100 | 29094 | 99 |
| 1074 | **+** | 35 | F | 478 | 10094 | 61883 | ND |
| 1075 | **+** | 31 | F | 965 | 5923 | 5546 | 100 |
| 1079 | **+** | 34 | F | 591 | 32485 | 26974 | 100 |
| 1119 | **+** | 30 | M | 545 | 580150 | 544444 | 98 |
| 1129 | **+** | 32 | F | 510 | 4559 | 5394 | ND |
| 1141 | **+** | 32 | F | 552 | 9826 | 2850 | ND |

CD4+ T cell count is in cells/μl. BAL viral load as determined by BAL viral load measured in cell-free fluid divided by ml epithelial lining fluid (ELF), which was calculated by multiplying the recovered BAL volume by BAL urea concentration/plasma urea concentration; QFT result, Quantiferon TB test result; % AMs before adherence, % of BAL cells that were alveolar macrophages (AMs) before adherence.

**Supplementary Fig 1. HIV is detectable in AMs from people with higher BAL HIV viral load despite low HIV receptor expression and T-tropic HIV infection rates.**

(**A**) HIV infected participants with undetectable HIV RNA in their AMs had lower BAL viral load than those with detectable HIV RNA in their AMs; BAL viral load was adjusted per ml of epithelial lining fluid (ELF). Clear circles denote values below the lower limit of detection (4 copies/ml), which was then adjusted to ELF. Statistics are from the Mann-Whitney test. (**B**) CD4 and (**C**) CCR5 were stained by flow cytometry and gated using fluorescence minus one controls on AMs, MDMs, and BAL T cells; n=13 for AMs and BAL T cells; n = 6 for MDMs. Comparisons were made with using Kruskal-Wallis with Dunn’s multiple comparisons test. (**D**) Cells were pre-treated for 1 hour with 40 μM of the CCR5 entry inhibitor maraviroc (MVC) or DMSO and viral entry was then assessed using gravity infection with BLaM-Vpr constructs for 12 hours with JR-CSF at a MOI of 1. HIV entry was detected by cleavage of a fluorescent BLaM substrate and measured by flow cytometry; n = 6, Wilcoxon matched-pairs test. (**E, F**) CD4+ T cells, MDMs and AMs were infected with replication-competent JR-CSF (**E**) or 89.6 (**F**) at a MOI of 0.2 for 12 hours. HIV p24 levels in the supernatant were measured by ELISA at the indicated time points; n = 11 for T cells, n = 12 for MDMs, and n = 6 for AMs. Statistics were done by Kruskal-Wallis tests with Dunn’s multiple test correction. * p<0.05, ** p<0.01, **** p<0.0001.

**Supplementary Fig 2. HIV *env* sequences from AMs, cell-free BAL fluid and plasma have variable compartmentalization.**

HIV *env* was isolated by PCR using conserved primers, cloned into pHDM plasmids, and sequenced on an Illumina MiSeq. Outlined arrows represent sequences used for cell-free experiments only; solid arrows represent sequences used for both cell-free (**Fig 2**) and cell-to-cell (**Fig 4**) experiments. Statistics are from the Nearest Neighbor statistic using the HYPHY package, comparing AM to plasma sequences within each HIV-infected donor.

**Supplementary Fig 3. AM- and plasma-derived HIV primary isolates are T-tropic.**

HIV *env* isolated from AMs or plasma from 3 HIV-infected donors was cloned into NL4-3 delta *env* backbone plasmids. Replication-competent virus was added to activated CD4+ T cells or MDMs for 12 hours and washed, and supernatant was collected 1 hour after washing (day 1) and after 7 or 14 days. Cells were infected with AM-derived viral isolates (**A**) from viral donor 1, 2 or 3 or plasma-derived viral isolates (**B**) from viral donor 1, 2 or 3. Data from all viral isolates from the same viral donor and anatomical site were pooled. CD4+ T cells and MDMs were isolated from the same six HIV-uninfected blood donors. Statistics by Kruskal-Wallis test. * p<0.05, ** p<0.01, *** p<0.001.

**Supplementary Fig 4. HIV infection in T cell-macrophage co-cultures is blocked by reverse transcriptase inhibition.**

MDMs were matured for 7 days or AMs were adhered for 1 hour. CD4+ T cells were activated with PHA for 3 days, infected with JR-CSF virus at a MOI of 0.2 overnight, washed, and cultured for 4 days, and then treated with 100 nM efavirenz (EFV) for 1 hour or DMSO control. HIV replication by SN or CTC conditions were assessed after 14 days. Supernatant from (**A**) MDMs or (**B**) AMs was collected and HIV p24 was measured by ELISA; n = 8 for MDMs; n = 6 for AMs; p value from Wilcoxon matched-pairs signed-rank test. * p<0.05.

**Supplementary Fig 5. Viral constructs made with patient-derived HIV *env* from AMs and plasma productively infect MDMs more efficiently through T cell contact.**

Experimental conditions were as in Fig 3 with the HIV constructs described in Fig 2. Cells were infected with AM-derived (**A**) or plasma-derived (**B**) viral isolates from viral Donors 1, 2 or 3. CD4+ T cells and MDMs were isolated from the same blood donors; n = 10. p value from Kruskal-Wallis test. ** p<0.01, *** p<0.001.

**Supplementary Fig 6. T-tropic HIV-infection of AMs is due to HIV transmission from Gag+ T cells to AMs.**

AMs were co-cultured with JR-CSF-infected autologous CD4+ T cells (cell-to-cell, CTC) or T cell supernatant (SN) for 3 hours, washed, and cultured and stained as described in Fig 5. (**A**) Cells were quantified and plotted for percent CD68+ Gag+ cells of CD68+ cells and compared with the Wilcoxon matched-pairs signed-rank test. Total Gag+ macrophages were increased in the CTC compared to the SN condition. (**B, C**) % Gag+ cells out of all nucleated cells were quantified and plotted. (**B**) AMs with associated CD3+ T cells with Gag staining only in the CD3+ T cell were not different in the CTC condition compared to the SN condition. (**C**) Gag+ AMs without associated T cells were not different between the SN and CTC condition. Each category was compared between SN and CTC by the Friedman test with Dunn’s multiple comparison’s test. 100 cells per donor were quantified; n = 6 donors. * p<0.05.

1. N. L. Michael *et al.*, Development of calibrated viral load standards for group M subtypes of human immunodeficiency virus type 1 and performance of an improved AMPLICOR HIV-1 MONITOR test with isolates of diverse subtypes. *J Clin Microbiol* **37**, 2557-2563 (1999).

2. M. S. Malnati *et al.*, A universal real-time PCR assay for the quantification of group-M HIV-1 proviral load. *Nat Protoc* **3**, 1240-1248 (2008).

3. A. B. Balazs *et al.*, Vectored immunoprophylaxis protects humanized mice from mucosal HIV transmission. *Nat Med* **20**, 296-300 (2014).
