# Supplementary figures and images for: T cell-tropic HIV efficiently infects alveolar macrophages through contact with infected CD4+ T cells
Source: Sci Rep. 2021 Feb 16;11:3890. doi: 10.1038/s41598-021-82066-x (PMC7886866; doi:10.1038/s41598-021-82066-x)

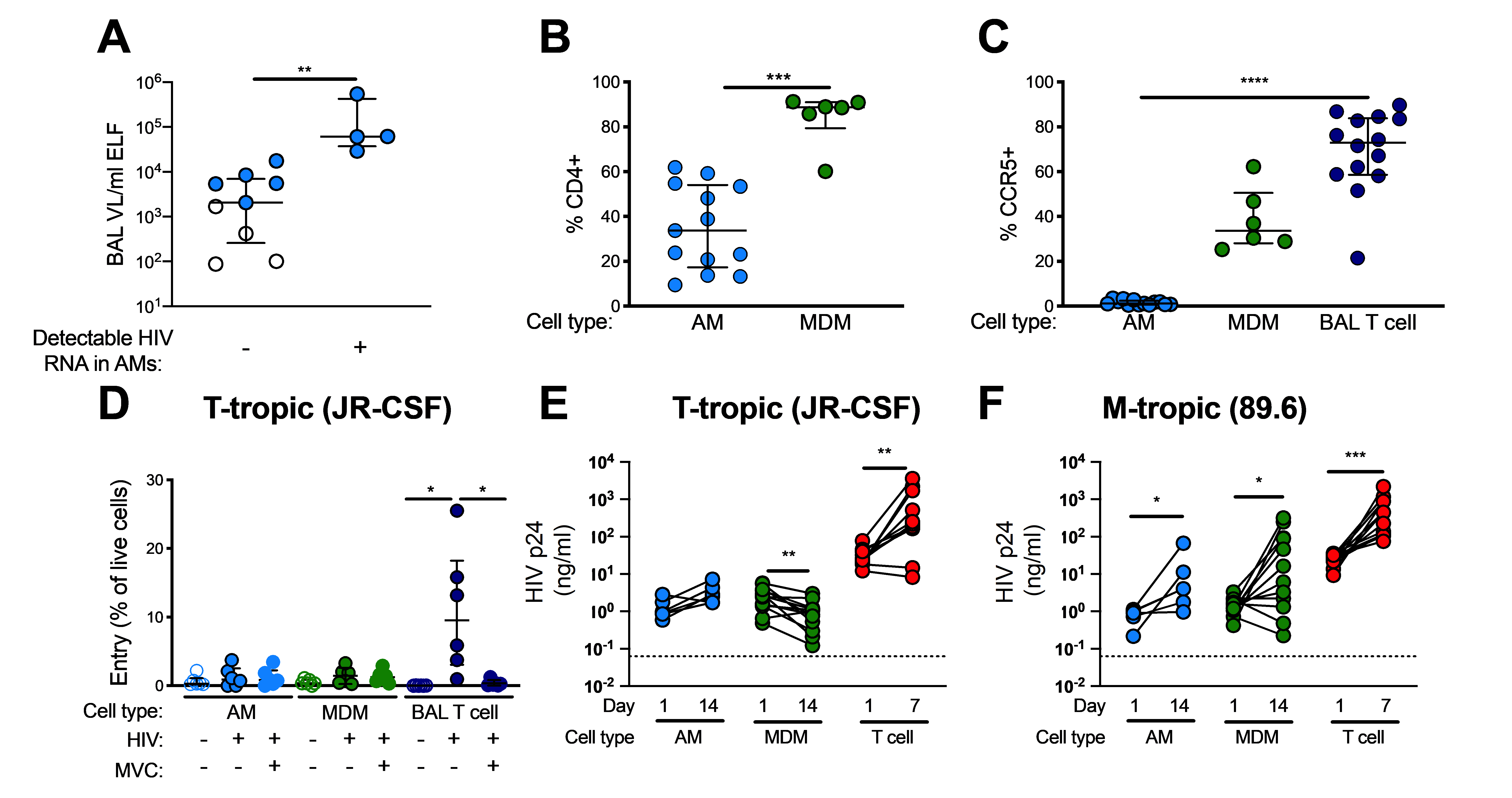

Supplement: Supplementary file 2 — Supplementary Figure 1. [file 41598_2021_82066_MOESM2_ESM.png]

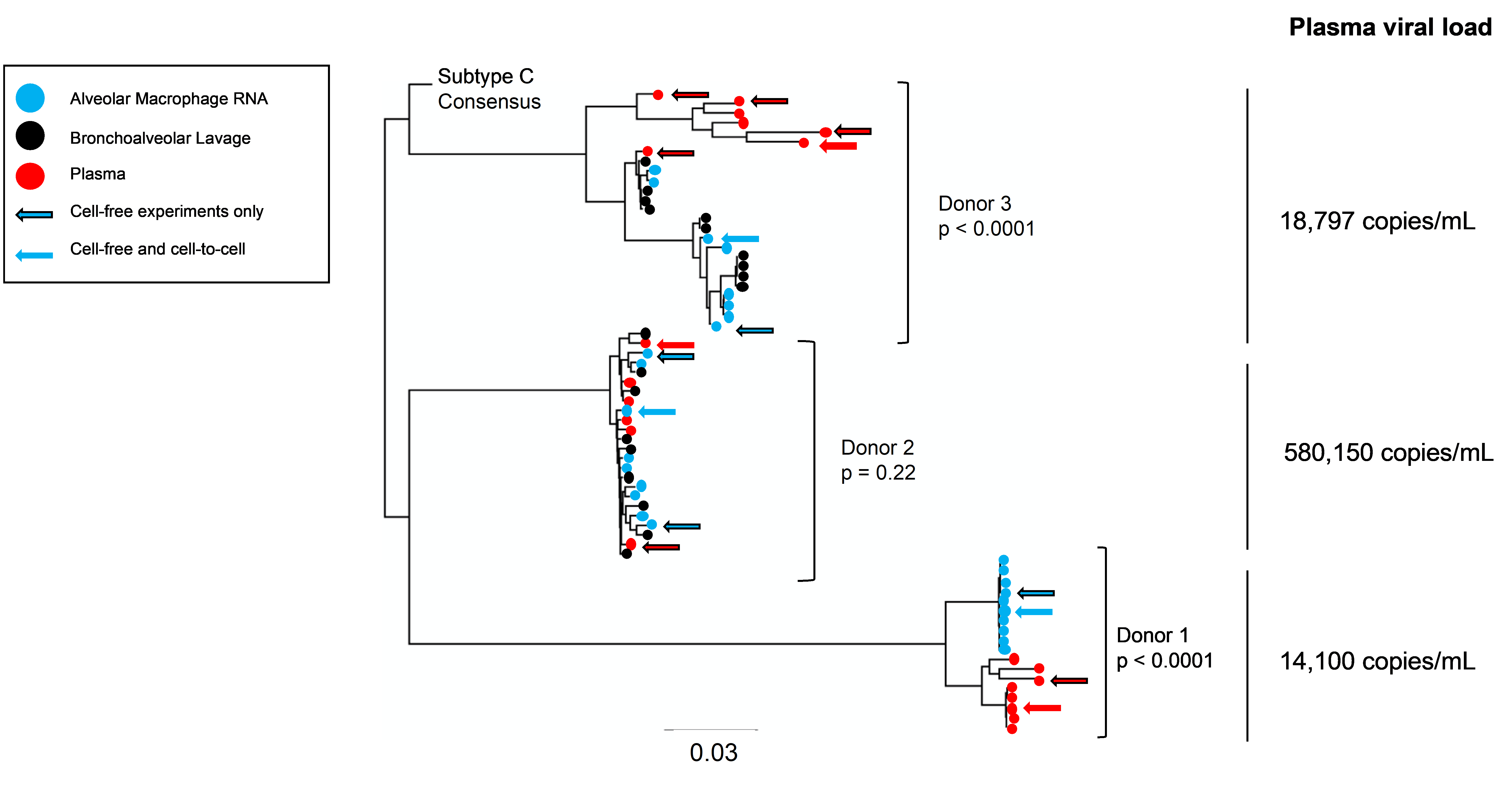

Supplement: Supplementary file 3 — Supplementary Figure 2. [file 41598_2021_82066_MOESM3_ESM.png]

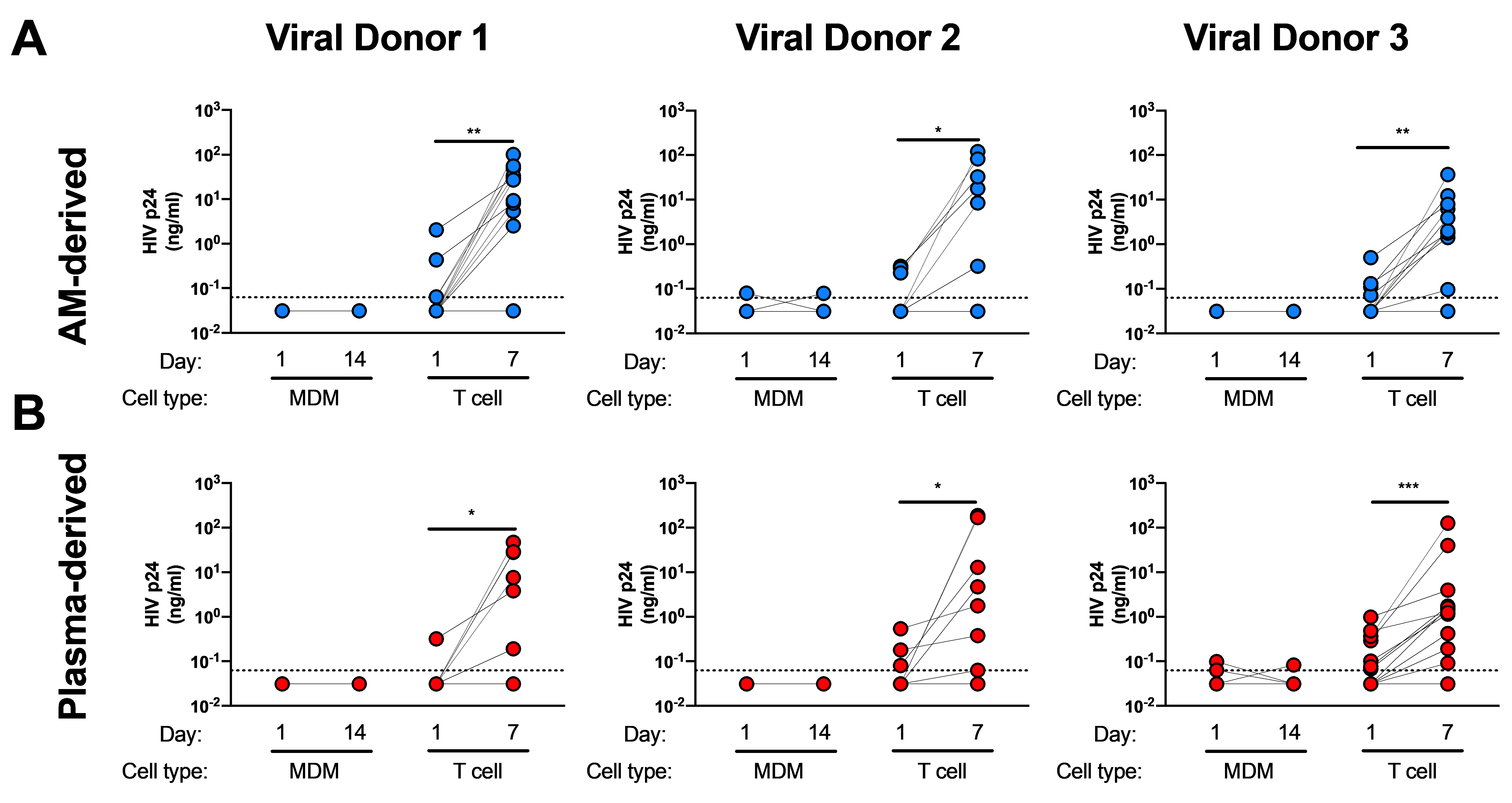

Supplement: Supplementary file 4 — Supplementary Figure 3. [file 41598_2021_82066_MOESM4_ESM.png]

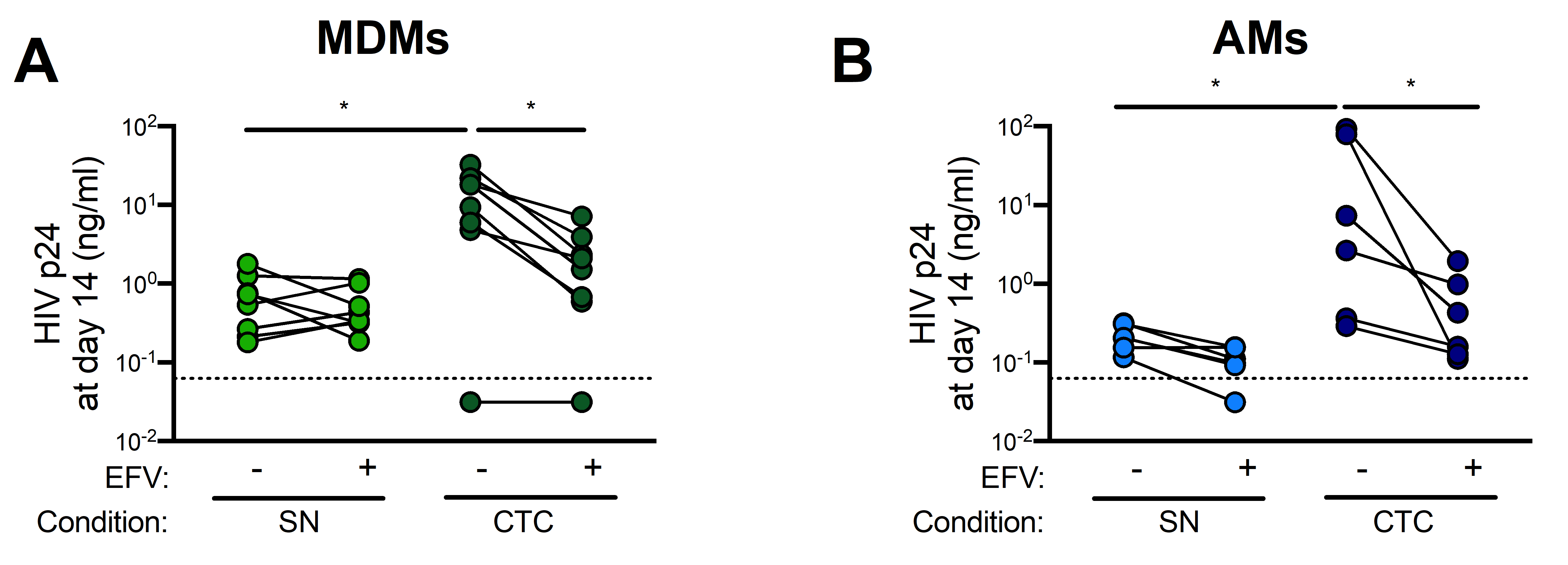

Supplement: Supplementary file 5 — Supplementary Figure 4. [file 41598_2021_82066_MOESM5_ESM.png]

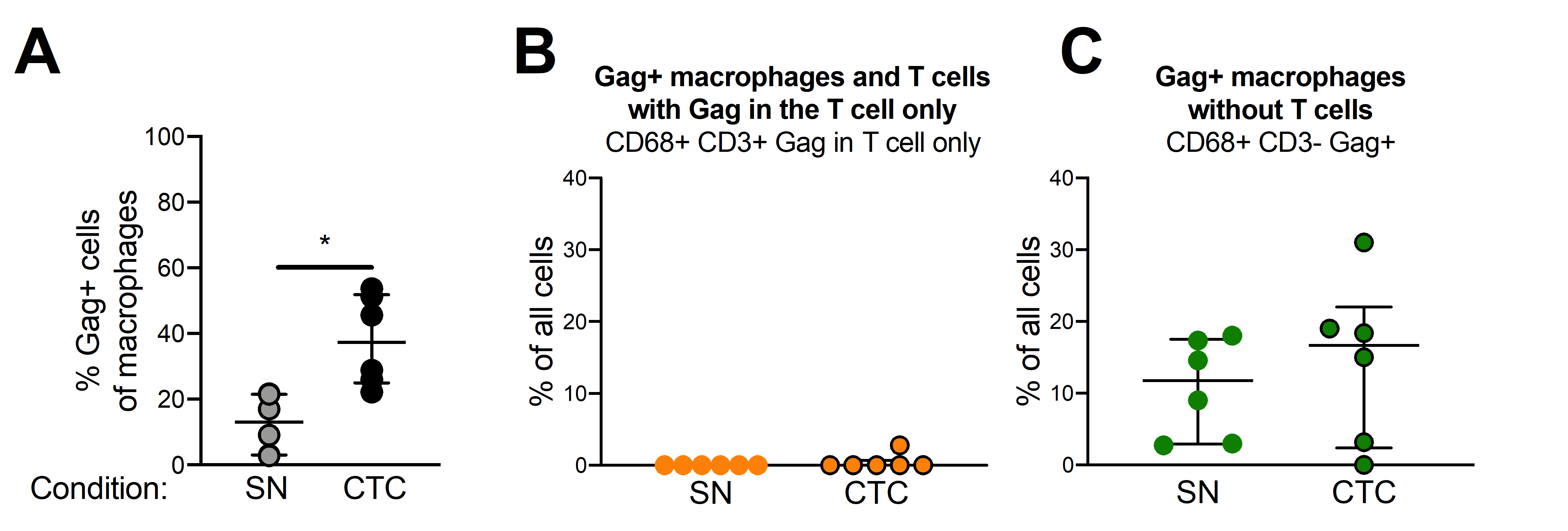

Supplement: Supplementary file 6 — Supplementary Figure 5. [file 41598_2021_82066_MOESM6_ESM.png]

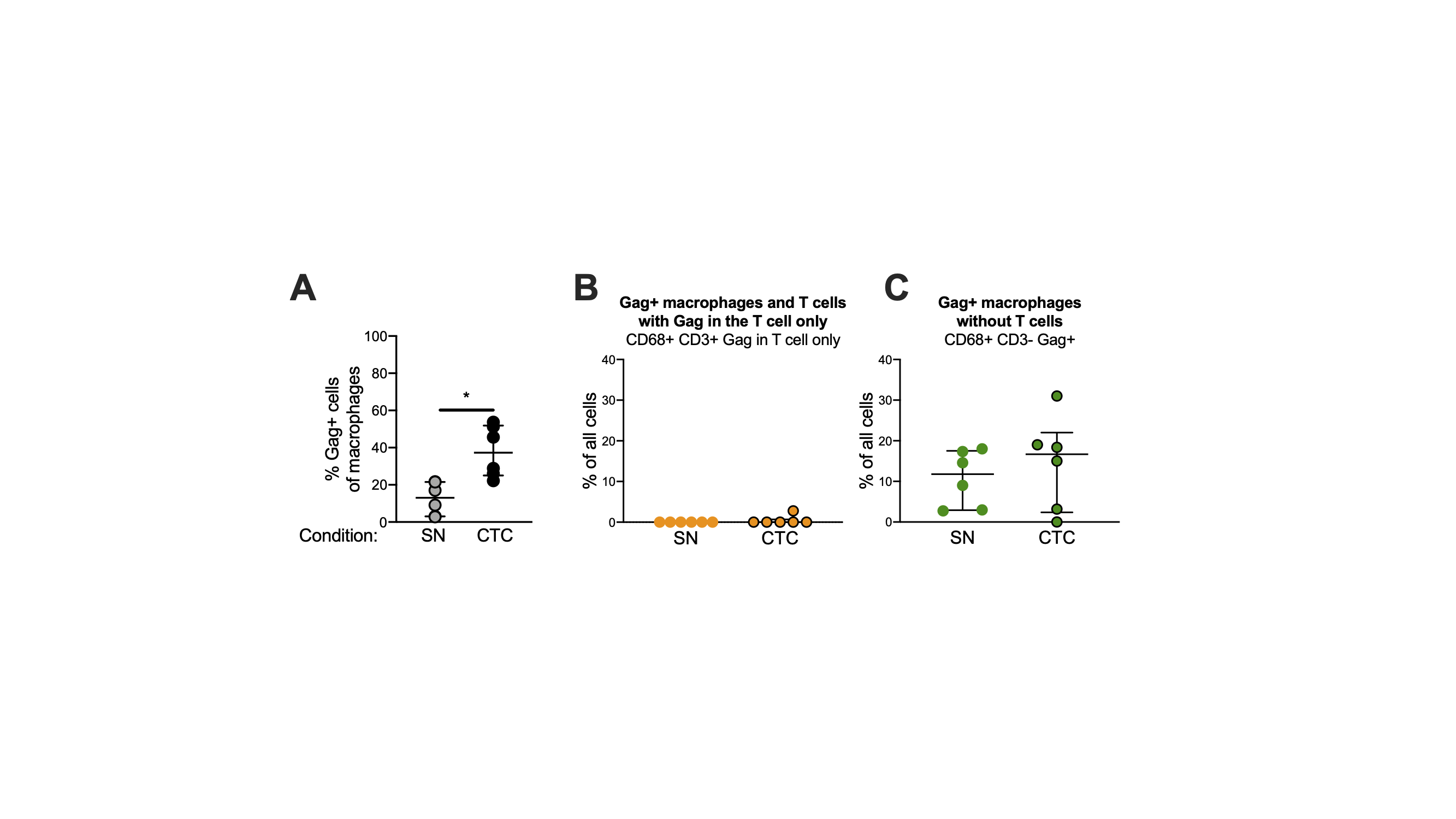

Supplement: Supplementary file 7 — Supplementary Figure 6. [file 41598_2021_82066_MOESM7_ESM.tiff]
